# Leveraging Polio Geographic Information System Platforms in the African Region for Mitigating COVID-19 Contact Tracing and Surveillance Challenges: Viewpoint

**DOI:** 10.2196/22544

**Published:** 2022-03-17

**Authors:** Godwin Ubong Akpan, Isah Mohammed Bello, Kebba Touray, Reuben Ngofa, Daniel Rasheed Oyaole, Sylvester Maleghemi, Marie Babona, Chanda Chikwanda, Alain Poy, Franck Mboussou, Opeayo Ogundiran, Benido Impouma, Richard Mihigo, Nda Konan Michel Yao, Johnson Muluh Ticha, Jude Tuma, Hani Farouk A Mohamed, Kehinde Kanmodi, Nonso Ephraim Ejiofor, John Kapoi Kipterer, Casimir Manengu, Francis Kasolo, Vincent Seaman, Pascal Mkanda

**Affiliations:** 1 Regional Office of Africa World Health Organization Brazzaville Congo; 2 World Health Organization Abuja Nigeria; 3 World Health Organization Juba South Sudan; 4 World Health Organization Geneva Switzerland; 5 School of Health and Life Sciences Teesside University Middlesbrough United Kingdom; 6 Bill and Melinda Gates Foundation Seattle, WA United States

**Keywords:** contact tracing, GIS, COVID-19, surveillance

## Abstract

**Background:**

The ongoing COVID-19 pandemic in Africa is an urgent public health crisis. Estimated models projected over 150,000 deaths and 4,600,000 hospitalizations in the first year of the disease in the absence of adequate interventions. Therefore, electronic contact tracing and surveillance have critical roles in decreasing COVID-19 transmission; yet, if not conducted properly, these methods can rapidly become a bottleneck for synchronized data collection, case detection, and case management. While the continent is currently reporting relatively low COVID-19 cases, digitized contact tracing mechanisms and surveillance reporting are necessary for standardizing real-time reporting of new chains of infection in order to quickly reverse growing trends and halt the pandemic.

**Objective:**

This paper aims to describe a COVID-19 contact tracing smartphone app that includes health facility surveillance with a real-time visualization platform. The app was developed by the AFRO (African Regional Office) GIS (geographic information system) Center, in collaboration with the World Health Organization (WHO) emergency preparedness and response team. The app was developed through the expertise and experience gained from numerous digital apps that had been developed for polio surveillance and immunization via the WHO’s polio program in the African region.

**Methods:**

We repurposed the GIS infrastructures of the polio program and the database structure that relies on mobile data collection that is built on the Open Data Kit. We harnessed the technology for visualization of real-time COVID-19 data using dynamic dashboards built on Power BI, ArcGIS Online, and Tableau. The contact tracing app was developed with the pragmatic considerations of COVID-19 peculiarities. The app underwent testing by field surveillance colleagues to meet the requirements of linking contacts to cases and monitoring chains of transmission. The health facility surveillance app was developed from the knowledge and assessment of models of surveillance at the health facility level for other diseases of public health importance. The Integrated Supportive Supervision app was added as an appendage to the pre-existing paper-based surveillance form. These two mobile apps collected information on cases and contact tracing, alongside alert information on COVID-19 reports at the health facility level; the information was linked to visualization platforms in order to enable actionable insights.

**Results:**

The contact tracing app and platform were piloted between April and June 2020; they were then put to use in Zimbabwe, Benin, Cameroon, Uganda, Nigeria, and South Sudan, and their use has generated some palpable successes with respect to COVID-19 surveillance. However, the COVID-19 health facility–based surveillance app has been used more extensively, as it has been used in 27 countries in the region.

**Conclusions:**

In light of the above information, this paper was written to give an overview of the app and visualization platform development, app and platform deployment, ease of replicability, and preliminary outcome evaluation of their use in the field. From a regional perspective, integration of contact tracing and surveillance data into one platform provides the AFRO with a more accurate method of monitoring countries’ efforts in their response to COVID-19, while guiding public health decisions and the assessment of risk of COVID-19.

## Introduction

Since the confirmation of COVID-19 in Wuhan, China, in late December 2019 [1], the disease continues to spread globally. The African region has not been spared [2]. As of July 5, 2020, the continent has recorded 466,300 cases and 11,121 deaths across all countries. Currently, the African continent, which makes up about 16.7% of the world’s population, accounts for 4% of the global cases [3]. Existing evidence shows that countries that have implemented public health measures, including rapid case identification, testing, isolation, contact tracing, and quarantine of contacts, at the onset of outbreaks have suppressed the spread of COVID-19 to low thresholds, ones that do not overwhelm existing health systems. In those countries, excess mortality has been prevented as they have been able to deliver quality clinical care and minimize secondary mortality due to other causes through the continuity of essential health services [4]. With no available vaccine or therapeutics, contact tracing, social distancing, and quarantine are the only available strategies for controlling the pandemic. In Africa, where testing capacity varies greatly and is very limited in most of its member states, the importance of contact tracing in stopping further progression of COVID-19 cannot be overemphasized.

Contact tracing is a process that involves early case recognition, isolation, and tracking of people who have been exposed to a disease [5,6]. It is an essential public health tool (Textbox 1 [7-11]) for breaking human chains of transmission and has been used extensively in the control of different types of infectious disease outbreaks. Recent studies on COVID-19 surveillance (Textbox 1) have used digital contact tracing combined with other measures, such as social distancing and quarantine, to demonstrate a greater effect on the reduction of new COVID-19 cases [12]. Pertinently, Wei et al [13] and Ng et al [14] demonstrated the public health importance of contact tracing on both asymptomatic and presymptomatic persons infected with COVID-19; these are considered silent drivers of COVID-19 infection.

The implementation of an efficient contact tracing system can vary depending on the place and the type of disease. In the case of COVID-19, for instance, controlling the epidemic with a comprehensive contact tracing system guides the type of intervention to be used (ie, self-monitored quarantine for presymptomatic and asymptomatic contacts for the duration of the incubation), whereas those with severe active disease may be hospitalized. Additionally, in most scenarios, simple traditional and manual contact tracing methods can be effective at the beginning of outbreaks when the numbers are low; however, large-scale epidemics and pandemics require newer digital contact tracing methods. During highly infectious pandemics, such as the current COVID-19 pandemic where very large populations are affected, it is pertinent to use digital solutions to effectively locate contacts who have been exposed to people with the disease as well as to monitor them consistently, especially those currently on home care and self-isolation.

Numerous types of contact tracing methods have been implemented during different pandemics and endemics, and their effectiveness largely depends on the tools used [15]. Geographic information system (GIS) technology and big data analytics are major tools used in contact tracing during large outbreaks. GIS technology had been used successfully in the past to identify the rate of transmission and corresponding incidences of notorious airborne diseases [16]. Also, big data analytics have been used successfully for real-time contact tracing of disease outbreaks in livestock, as well as with other highly contagious viral respiratory diseases, such as SARS and fibromuscular dysplasia [17,18].

Over the years, scientific data collated by the World Health Organization’s (WHO) emergency preparedness and response (EPR) unit showed that most countries in Africa are struggling to implement efficient contact tracing methods.This has greatly increased the number of new community-transmitted COVID-19 cases; this is a very critical situation for the continent, as it has been predicted to be the next hot spot for the coronavirus [19]. To effectively curb the problem of inefficient contact tracing in Africa, the WHO has sought the support of the African Regional Office (AFRO) GIS to build a GIS-enabled tool for contact tracing that is pertinent to the African continent. Since 2017, the AFRO GIS Center has put in place mobile-based solutions to collect health information in near real time and, thus, has access to health program implementation and can measure the effectiveness of interventions. The tools provided by the center contributed significantly to the successes recorded with polio certification efforts [20]. In the course of proferring a workable solution, the AFRO GIS Center developed an application model (Textbox 1)—a GIS-enabled tool—for contact tracing for COVID-19 surveillance. In this paper, we present a robust and efficient contact tracing app that can be used across Africa to effectively respond to the COVID-19 outbreak. This app builds on effective tools, such as the Open Data Kit (ODK) [21], to collect and manage data in a constrained environment, combined with the AFRO Polio GIS platform (Textbox 1) [22], in order to locate, identify, monitor, and track contacts during the COVID-19 pandemic or any other large-scale pandemic. However, before proceeding to the description of the app, it will be of relevance to give an overview of a very important COVID-19 surveillance problem in Africa: the problem of contact tracing, in which the quest for solving this particular problem led to the development, deployment, and use of the app in Africa.

**Definition of selected terms.**

Application: a program or group of programs designed for end users. Examples of an application include a word processor, a spreadsheet, an accounting application, a web browser, an email client, a media player, a file viewer, a simulator, a console game, or a photo editor [7].

Platform: a computing platform or digital platform is the environment in which a piece of software is executed. It may be the hardware, the operating system, a web browser and its associated application programming interfaces, or other underlying software, as long as the program code is executed with it [8].

Module: a section of an application or app that focuses on a unique set of deliverables or assessments [9].

Surveillance: an epidemiological practice by which the spread of disease is monitored via data collection in order to establish patterns of progression. The main role of disease surveillance is to predict, observe, and minimize the harm caused by outbreak, epidemic, and pandemic situations, as well as to increase knowledge about which factors contribute to such circumstances. A key part of modern disease surveillance is the practice of disease case reporting [10].

Tool: a programming tool or software development tool is a computer program that software developers use to create, debug, maintain, or otherwise support other programs and applications [11].

## Effective Contact Tracing: The African Problem Regarding COVID-19 Surveillance

Contact tracing is a very serious limitation in disease surveillance in Africa. As shown in Figure S1 in Multimedia Appendix 1, the problems associated with contact tracing in many African countries include an inadequate number of skilled personnel who are already overstretched, suboptimal technology and tools, underfunded health systems, and poor infrastructure with associated myths, rumors, and communication barriers.

## WHO AFRO–Recommended Solutions for Contact Tracing in Africa: Key Points

### Overview

The WHO’s AFRO GIS Center considered different scenarios during preliminary discussions in March 2020, with the COVID-19 incident management team managing the regional response at WHO’s regional office. During these preliminary discussions, it was highlighted that the best contact tracing solution would leverage existing GIS platforms and would be deployed to fill the current gaps known in COVID-19 surveillance and to help address existing challenges with contact tracing (Figure S1 in Multimedia Appendix 1).

It was noted that while traditional contact tracing could still work in places with few contacts, these approaches would be constrained in countries with many contacts, coupled with the impact of lockdowns on contact tracing [23]. Consequently, the technical team that was tasked to review different possible solutions to the problems associated with contact tracing in Africa recommended the following:

1. The rapid development of a GIS-enabled COVID-19 self-reporting contact tracing app.
2. The rapid deployment of a contact registration and follow-up app by COVID-19 surveillance teams in-country.
3. Visualization of field surveillance and reporting with interactive and near real-time dashboards.
4. COVID-19 surveillance at the health facility level to assess the preparedness and readiness of health systems to cope with COVID-19 at this reporting level.

Following the review, Benin and Zimbabwe were selected based on their indication of interest to immediately use some or all aspects of the platform.

### The Rapid Development of a GIS-Enabled COVID-19 Self-reporting Contact Tracing App

#### Overview

At the very early stage of the COVID-19 outbreak in Africa, the AFRO GIS Center leveraged the success of its contribution in the eradication of polio in the African region. Coupled with its surveillance experience over the years [24] and in collaboration with the WHO EPR team, the AFRO GIS Center was able to develop some novel tools for immediate field deployment to collect real-time data and monitor the COVID-19 pandemic in the field by surveillance personnel. Different technologies were used to develop and integrate this rapid intervention (ie, the COVID-19 app used by surveillance personnel); these included the following: ODK technology [25] and stacks via Ona, which is an open-source tool. These tools are housed and secured inside the WHO infrastructure, primarily for real-time data collection in the field; these include the use of external tools like ArcGIS Online [26], Power BI [27], application programming interfaces (APIs), KoBoToolbox [28], and DHIS2 (District Health Information Software 2) [29] for contact tracing and visualization processes. ArcGIS Online and Power BI are data visualization platforms that have different use cases for real-time data visualization.

Shortly after the development and deployment of the above-described application for COVID-19 surveillance personnel, the AFRO GIS Center went ahead to develop a GIS-enabled COVID-19 self-reporting contact tracing app for registering and following up contacts by the surveillance teams in-country, in accordance with the recommendations of the COVID-19 incident management team of the WHO AFRO. The developed app enables contacts in home-based care, self-isolation, and in quarantine centers to provide daily updates on their health condition. In addition, the app provides an opportunity for these contacts to be able to identify the nearest health facility to which they can quickly report if they develop any symptoms of the disease. Importantly, the traditional in-country contact tracing teams are also able to register and follow up contacts and cases with the app or any other app that the country may opt for. If the country opts for other apps, the solution allows for interoperability of the toolbox, which enables aggregating, analyzing, and visualizing of the data in the same regional dashboards.

Figure S2 in Multimedia Appendix 1 depicts the architecture of the COVID-19 self-reporting contact tracing app developed by the AFRO GIS Center, in collaboration with the WHO EPR team. The app has the following three components: (1) a data collection component, (2) an API component, and (3) a polio GIS toolbox and platform.

#### Data Collection Component

##### Overview

The architecture was structured to accommodate a wide range of data collection tools, giving countries the liberty to use the WHO data collection tool or any data collection tool that best suits them, such as KoBoToolbox and DHIS2. These data collection tools are then imported in real time into the polio GIS platform for analysis and visualization. These tools are summarized in the following sections.

##### COVID-19 Self-report Form

**Figure 1:**
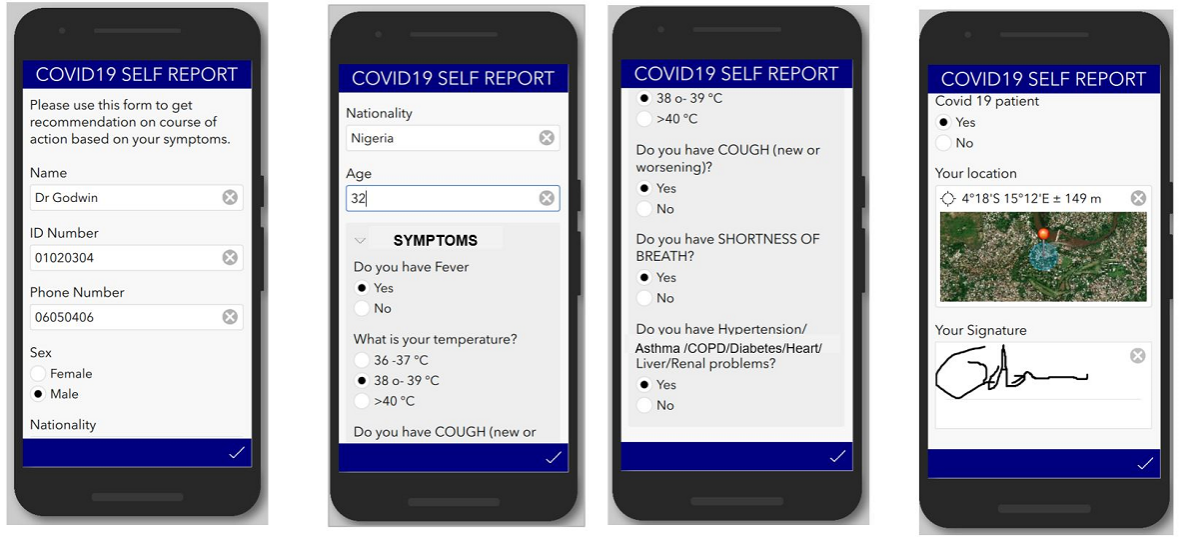
An example of a self-report form for use with widespread contacts.

This form was structured to collect details and the daily status of a case or contact under quarantine or being followed up for the mandatory 14-day period. It enables the individual to self-report his or her daily status and submit to the central servers for monitoring and feedback (Figure 1).

##### COVID-19 Case Investigation Form

This form was structured to collect the details and profiles of confirmed, suspected, and probable cases. The form can also collect the medical provider’s information, patient information, clinical information, travel history, and final classification of the case. It is important to note that this form is linked to the contact listing form; as such, the case phone number or a concatenation of the country, province, district, and case number is used as a unique identifier for referencing and following up. This form is used by health personnel at the health facility level once a COVID-19 case is suspected.

##### Contact Listing and Follow-up Form

This form is divided into two components: the first component is used to collect the contacts of a confirmed case (ie, contact tracing form), and the second component is used to collect and record the temperature reading of a case contact for 14 days (ie, contact follow-up and self-reporting). This form references the actual case for those contacts that could be linked to a COVID-19 case.

##### Contact Registration Form

Every contact connected to a case is expected to be registered in the COVID-19 database. A prompt is shown at the beginning of the form that allows for contact registration. The index case’s ID number is required at this stage to link the contact to an already-existing case in the database (ie, a new contact cannot be recorded without a prerecorded index case in the database). This displays the records of the index case for confirmation and validation before registering the details of the new contact.

##### Contact Follow-up Form

After contact registration, the contacts are expected to have a 14-day record update of their temperature and medical condition. There is a prompt at the beginning of the form that allows for contact follow-up (ie, it prompts self-isolated contacts to submit daily results for 14 days).

##### Traveler Health Questionnaire and Follow-up Form

This form is divided into two components: the first component is used to collect general information about a traveler entering into the country, and the second component is used to collect and record the temperature reading of the traveler for 14 days (ie, contact follow-up). Note that the follow-up component is linked to the traveler health questionnaire and is referenced using the case phone number of the traveler as the unique identifier.

#### API Component

This component is responsible for the interoperability and interaction of data between platforms. The APIs link the various platforms together, facilitating data exchange in real time. This allows for seamless operations and data exchange between the data collection, hosting, analytics, and visualization tools within the polio GIS toolbox and platform.

#### Polio GIS Toolbox and Platform

This comprises all the resources developed and deployed within the WHO AFRO infrastructure. It comprises front-end data collection tools on mobile devices and web interfaces, database servers, data visualization tools, and platforms.

### Rapid Deployment of the Self-reporting Contact Tracing App

The AFRO GIS Center, in collaboration with the WHO EPR team, went ahead to deploy the COVID-19 Emergency Deployment Toolkit, using the ODK platform to support contact tracing through self-reporting.

### Contact Tracing Data Management

#### Overview

Data management is a very crucial aspect of surveillance, particularly when it comes to data of relevance to contact tracing. In the management of contact tracing data, database linkages, performance monitoring, visualization, and analysis are important factors to consider. How these factors were managed are discussed below.

#### Database Linkages

**Figure 2:**
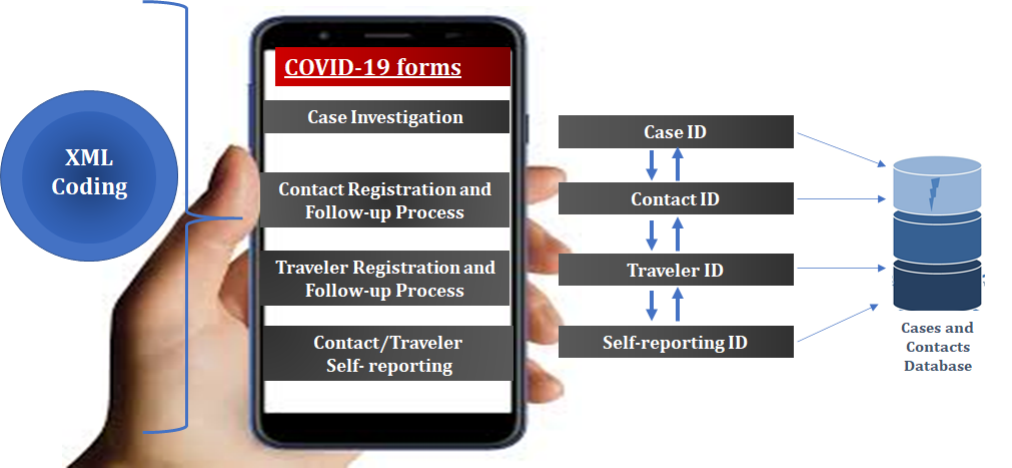
African Regional Office geographic information system architecture showing forms.

The biggest challenge of contact tracing data management is enrolling contacts and performance monitoring of the contact tracing processes [23]. This was overcome by ensuring that each case ID was matched to the contact ID and that the traveler ID was listed in the contact database during the quarantine period for ease of tracking if the traveler were to become a case. This section describes the linkage system we used to connect the identity management system of the cases to contacts and travelers, while maintaining confidentiality and integrity of the cases and our contacts database (Figure 2).

#### Linking the Dynamic Case and Contact Data via XML Form to Populate the Case and Contact Database

**Figure 3:**
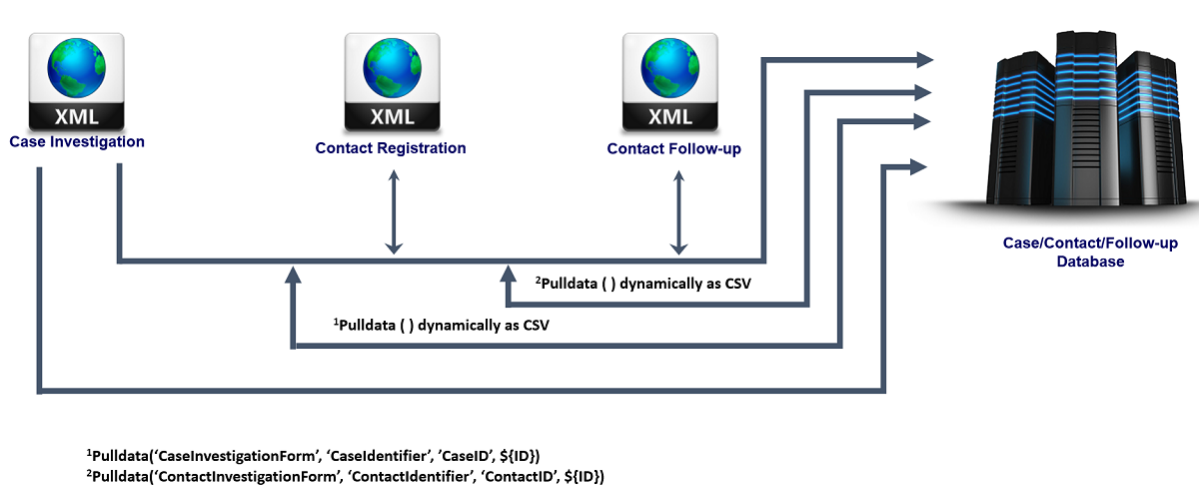
Linking dynamic data via XML files using pull functions.

This process hinges around specifying form data as a media file for another form. Used in conjunction with the *pulldata* function from the ODK, this allows the developer to pull data from other dynamic data sets and surveys (ie, other forms in the Ona ODK system that are still active and accepting submissions) in the same or different project, similar to pulling data from a preloaded CSV file (Figure 3).

On clicking the button “Link Dataset,” the changes are saved automatically. The linked data set appears in forms on the Android device. After data set linkage to the form, data can be effectively pulled from the linked data set into the form using the *pulldata* function under the calculation column. The file name of the linked data set entered above will be the file name referenced in the *pulldata* function (ie, locations). See Figure 3 for an illustration of the *pulldata* function and considerations for replicating this in another situation that warrants the use of dynamically pulled data from one mobile form through a server to another mobile form.

#### Performance Monitoring

Evidence-based performance monitoring of contact tracing, surveillance activities, and other health interventions by health workers was a key consideration in the repurposing of polio GIS platforms. A geotracking feature was added to our app to provide geographic evidence of a team’s visits to specific contacts. This also ensures that health workers conduct surveillance and other health interventions with the knowledge that their activities are being monitored for accountability.

#### Visualization and Analysis

A one-stop dashboard that brings data from participating countries into a regional interface for COVID-19 activities was developed using connectors from the databases to ArcGIS and Power BI to develop the key performance indicators for the pillars of the COVID-19 response. The dashboard allows the incident management team to conduct an integrated and effective response to the pandemic. An example is seen in Figure S3 in Multimedia Appendix 1.

#### Health Facility–Based COVID-19 Surveillance

A module (Textbox 1) for reporting COVID-19 activities, infection prevention and control (IPC) readiness assessment, and recording of cases that fit the COVID-19 clinical definitions in facility-based registers was developed and deployed for field use by health workers that conduct surveillance at the facility level. This module leveraged an existing intervention called the Integrated Supportive Supervision (ISS) app. Thus, it was easy to deploy, as the development was appended on top of the existing app, which is in use by over 5000 health workers across the region with supportive supervision visits that average 150,000 annual visits across the African region.

The module focused on the use of the ODK-based app (ie, the ISS app) with the following COVID-19 assessment focuses:

1. Awareness of the existence of a COVID-19 surveillance system.
2. Display of COVID-19 case definition posters.
3. Presence of a COVID-19 surveillance focal point.
4. Number of health workers in the facility who know the definition of a suspect case.
5. Number of people who know the COVID-19 alerting number and notification channel.
6. Appraisal of IPC awareness in terms of handwashing, functioning of handwashing kits, availability of isolation room, use of personal protective equipment, and availability of a triage system in the health facility.
7. Recording of details of suspected cases that fit the clinical system of COVID-19 and facilitating testing.

## Field Use and Outcome Evaluation

### Contact Tracing Use

The adoption of the solutions developed by the AFRO GIS Center in collaboration with the WHO EPR team was piloted in Zimbabwe and Benin in April 2020; the solutions were then subsequently used in Nigeria, Uganda, Cameroon, and South Sudan to conduct contact tracing or support other contact tracing data collection expeditions. See Figure S4 in Multimedia Appendix 1 for the status of deployments. Since most countries in the region (98%) are familiar with the deployment of the ODK tool from its use in polio eradication, the COVID-19 contact tracing app is quite easy for countries to deploy and use without extensive training. In fact, several additional countries have demonstrated interest following a conference call with the AFRO GIS Center, in which 15 countries participated. Countries already using similar tools for contact tracing were encouraged to share their APIs with AFRO to enable the GIS Center to pull data into the regional platform. However, countries with a high number of contacts have been encouraged to use the self-reporting module for daily contact self-reporting.

### Health Facility–Based COVID-19 Surveillance

**Figure 4:**
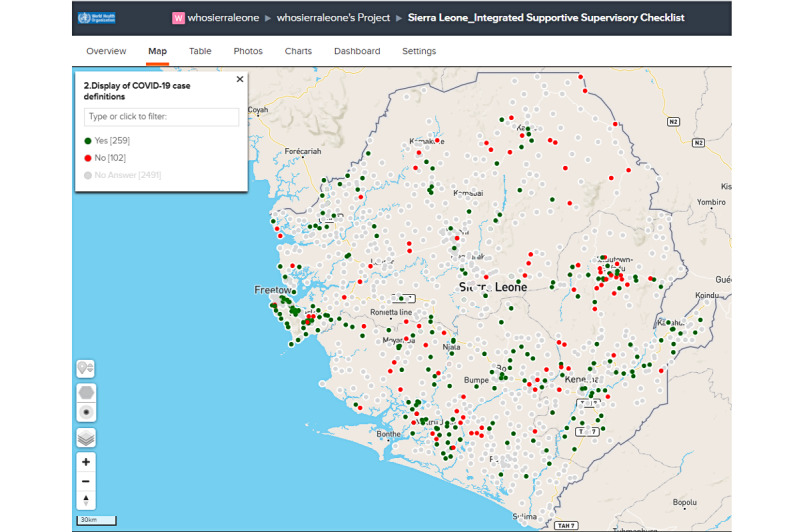
Sample status of COVID-19 sensitization posters and displays from the health facility–based surveillance module for Sierra Leone.

A total of 27 countries in the region adopted this module, which was developed to mitigate the gaps of COVID-19 surveillance at the health facility level. Event-based surveillance is highly optimized in low- and middle-income countries at the health facility level [30,31] and could also be key to improved COVID-19 surveillance. This module was easy to develop and deploy, as there was no training necessary since other priority disease surveillance was already ongoing at the health facility level using the ODK-based ISS forms. Real-time interactive visualization of the data from health facility–based surveillance is available at the country level for all the key variables. Also, geographic representation in maps for important accessories for IPC and triaging by the health facility is accessible to decision makers at all levels in-country. Figure 4 shows an example of a map of a health facility with COVID-19 posters displayed with definitions, which is an entry point for adequate sensitization at that level of reporting.

## Discussion

### Strengths of the Polio GIS Platform for Contact Tracing and Health Facility–Based Surveillance

The scope of leveraging the polio GIS platform in terms of apps and visualization tools addresses the mitigation plans for contact tracing and surveillance gaps in the region regarding the following aspects:

1. During the COVID-19 pandemic, the platform was able to support the identification of COVID-19 cases, contacts, and database consolidation gaps (eg, matching contacts to their index cases), helping to guide the response.
2. During outbreaks and routine contexts alike, the platform was able to identify community transmissions that were not detected by the traditional contact and case databases.
3. Health facility–based surveillance was built on an already-existing ISS module and was, thus, quite sustainable, as other active disease surveillance was already being conducted with the app.
4. The chain of transmission and surveillance gaps were easily seen on the interactive visualization made possible by the real-time connection of data being submitted from the apps into Power BI; thus, faster decision-making was possible at all levels.
5. The platform allowed ease of use of the data entry modules; there was little or no training, as surveillance teams at the country level were already used to similar technologies as a result of using mobile phones for other interventions.
6. The platform was developed with interoperability being given the highest consideration so that other countries’ contact tracing efforts and facility-based surveillance systems, such as DHIS2, KoBoTool, and SORMAS (Surveillance Outbreak Response Management and Analysis System), could be easily connected to share data.

This platform offers an additional dimension to the existing platforms that are being used in supporting the COVID-19 response [32,33], with features that make it more adaptive to the local context. In the African region, 93% of the countries are already using this platform for other health projects. The AFRO GIS Center has been leveraging GIS technology to ensure equitable access to essential health services, ranging from ISS for the Expanded Programme on Immunization, support for microplanning, effective coordination during the Ebola response, and monitoring the cholera outbreak response, among others [34,35], thereby making it easy to adapt and implement. The tool has been shown to support polio vaccination activities in complex humanitarian settings, including within refugee camps and camps for internally displaced people [36].

The use of the GIS platform has been shown to provide adequate support and promote care of individuals across different thematic areas of health. Documented evidence exists that has demonstrated its capability to identify high-priority areas that require maternal care [37]. In addition, it has helped to identify health trends, including tracking the spread of infectious diseases, such as Ebola and measles. The use of the GIS platform has been shown to help identify the problem, identify where it exists, and equally support the provision and maintenance of care for individuals through a more efficient and coherent manner.

### Trade-offs for Leveraging the Polio GIS Platform

Many questions with regard to information sensitivity and privacy regarding COVID-19 cases and contacts have been answered by the security features of the app and the platform. However, there still exists the possibility of intrusion, careless handling of passwords, and hacking that may lead to compromised information if countries do not adequately manage their access and control. Also, the app and platform deployment involves users having phones for data entry and visualization; thus, a lot of phones are required for implementation of all aspects of surveillance and contact tracing.

### How to Implement the Platform

The solutions outlined can easily be implemented with the procedural steps that were enumerated and explained in the data collection and visualization sections. The use of technology stacks via ODK and Power BI to collect data and to visualize them in real time can easily be replicated by technical users who are familiar with these tools. However, it is important to note that early engagement and meetings with all stakeholders was crucial to ensure ownership, increase coordination, and gain a better understanding of the existing surveillance landscape [31]. Good linkage to the response was described as essential for all systems, as every verified case and contact is documented. In order to ensure timely reporting of cases found via health facility–based reporting, health workers at the facility level required training and supportive supervision by trained district-level teams.

### Conclusions

Health facility–based surveillance and effective contact tracing management tools outlined here will provide valuable information that can strengthen the use of data by national surveillance systems during the pandemic. Evidence from the global pandemic thus far clearly presents three challenges in controlling COVID-19: its lasting pandemic potential, high fatality due to its infectivity, and its ability to disrupt health systems. Similarly, in the absence of vaccines and therapeutics, the only available tools for control include contact tracing, social distancing, and quarantine. The African region must, therefore, adopt a variety of methods to minimize the above challenges and adapt the response to the specific needs of each country as the outbreak evolves.

Digital contact tracing and surveillance at the facility level will be paramount at some point for every country to stay a step ahead of the virus. The applications and apps that have been developed for COVID-19 surveillance and contact tracing are not standalone interventions but should be implemented together with social distancing measures and quarantine, depending on the size of the outbreak.

In the African region, COVID-19 testing capacity varies widely. Countries with limited testing capacities and large outbreaks will need more advanced comprehensive contact tracing solutions, such as the one described herein, to suppress the virus to lower transmission rates. However, those with smaller outbreaks can use traditional methods with ODK or KoBoCollect platforms and simply share their APIs with the regional office to ensure that all data are available to the incident management system.

As countries begin to relax public health lockdowns, traditional and advanced contact tracing methods will be necessary to highlight areas of ongoing transmission; these data will be needed not only in their respective countries but also on a regional level to enable a better understanding of the pandemic and optimal decision-making in managing the risks and responses.

If widely adopted in the region, this innovation, alongside existing data collection tools (eg, KoBoCollect and DHIS2), will help countries respond effectively and efficiently to the pandemic. Other benefits to countries using this tool include real-time monitoring of the epidemic and their response, timeliness and completeness of contact tracing, and staff accountability. These factors are the cornerstone of surveillance during epidemics. During the Ebola outbreak in West Africa from 2014 to 2016, it was well-documented that a major contributor fueling the epidemic was the lack of standardized and synchronized contact tracing. Once adopted and implemented, a significant decrease in cases was observed across the affected countries, even though, at the time, the solutions were not as advanced as they are today.

With the high penetration rate of mobile phones across the African region, mobile-based monitoring of COVID-19, from traditional methods to voluntary self-reporting and remote follow-up of contacts, will greatly improve the identification of suspected cases and contacts; these are important resources to help in the region’s fight against this debilitating disease. Additionally, the use of this tool should reduce the burden on health systems, allowing for the provision of essential health services and minimizing mortalities from COVID-19 and neglected secondary diseases, which can result from a system overwhelmed by the pandemic.

From a regional perspective, integration of contact tracing and surveillance data into one platform provides the AFRO with a more accurate method of monitoring country efforts in their response to COVID-19, while guiding public health decisions and the assessment of risk for COVID-19.

## Appendix

Multimedia Appendix 1 Leveraging Polio GIS platforms in the African Region for mitigating Covid-19 contact tracing and Surveillance challenges.

## Abbreviations

AFRO: African Regional Office
API: application programming interface
DHIS2: District Health Information Software 2
EPR: emergency preparedness and response
GIS: geographic information system
IPC: infection prevention and control
ISS: Integrated Supportive Supervision
ODK: Open Data Kit
SORMAS: Surveillance Outbreak Response Management and Analysis System
WHO: World Health Organization
