# Supplementary material for: Leveraging Polio Geographic Information System Platforms in the African Region for Mitigating COVID-19 Contact Tracing and Surveillance Challenges: Viewpoint
Source: JMIR Mhealth Uhealth. 2022 Mar 17;10(3):e22544. doi: 10.2196/22544 (PMC8972111; doi:10.2196/22544)
Supplement: Multimedia Appendix 1 [file mhealth_v10i3e22544_app1.docx]

**Multimedia Appendix 1**

**Figure S1.** Challenges identified to be associated with COVID-19 surveillance, and their solutions


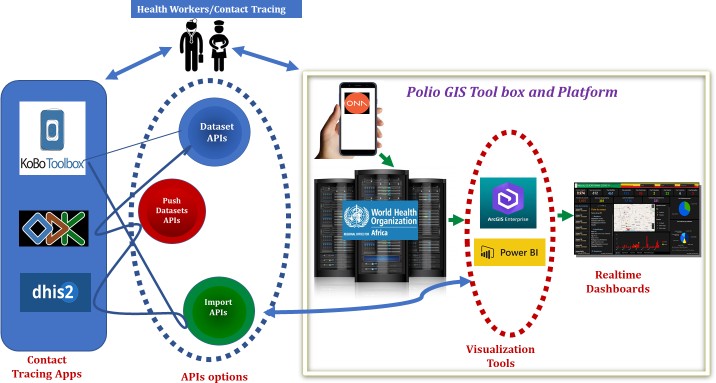


**Figure S2.** Architecture of the AFRO GIS centre’s model of the COVID-19 GIS-enabled Self-Reporting Contact Tracking Application


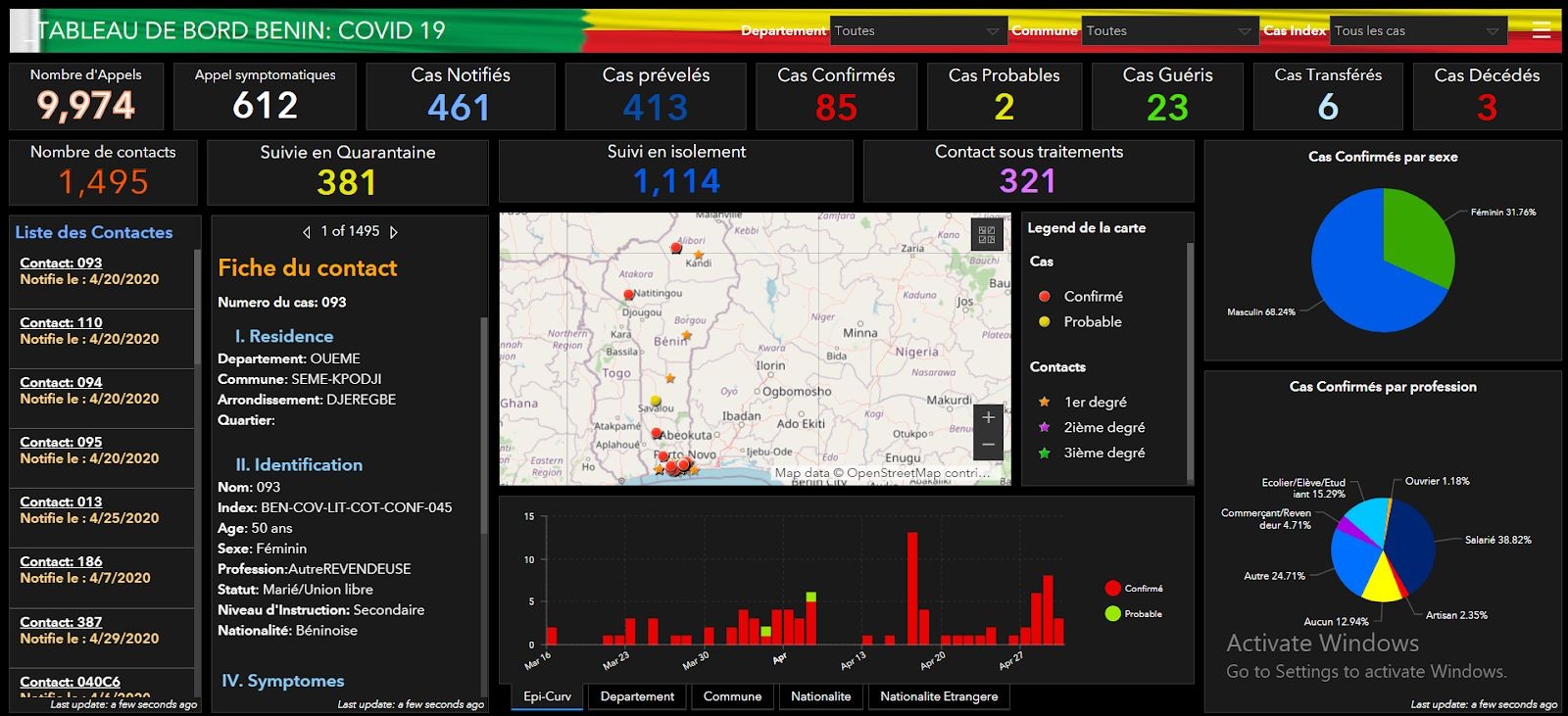


**Figure S3.** Sample Live dashboard aggregating data from Pillars of the Covid-19 Response


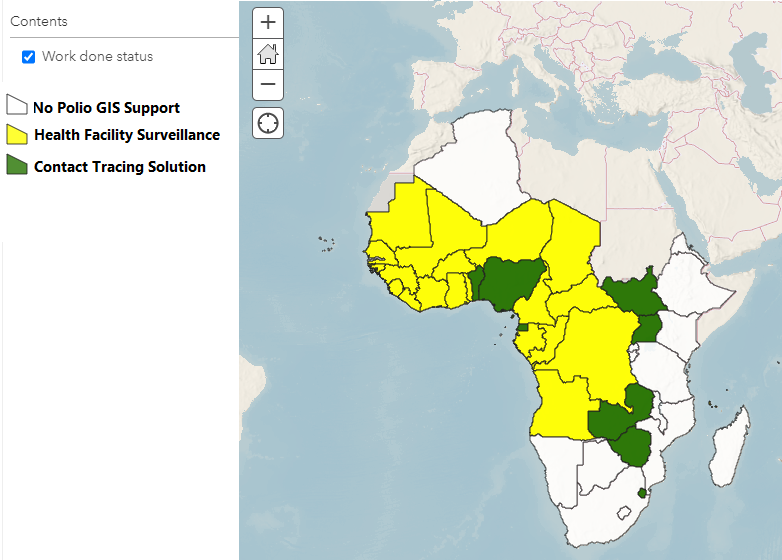


**Figure S4.** Status of Deployment and Use of the Polio GIS system for COVID-19 contact tracing and Health Facility based Surveillance
